# Wildlife health capacity enhancement in Thailand through the World Organisation for Animal Health Twinning Program

**DOI:** 10.3389/fvets.2024.1462280

**Published:** 2024-08-21

**Authors:** Sarin Suwanpakdee, Nareerat Sangkachai, Anuwat Wiratsudakul, Witthawat Wiriyarat, Walasinee Sakcamduang, Peerawat Wongluechai, Choenkwan Pabutta, Ladawan Sariya, Waruja Korkijthamkul, David S. Blehert, C. LeAnn White, Daniel P. Walsh, Craig Stephen, Parntep Ratanakorn, Jonathan M. Sleeman

**Affiliations:** ^1^Faculty of Veterinary Science, Mahidol University, Nakhon Pathom, Thailand; ^2^U.S. Geological Survey, National Wildlife Health Center, Madison, WI, United States; ^3^U.S. Geological Survey, Montana Wildlife Cooperative Unit, Bozeman, MT, United States; ^4^McEachran Institute, Nanoose Bay, BC, Canada; ^5^U.S. Geological Survey, St.Paul, MN, United States

**Keywords:** capacity enhancement, One Health, Twinning Project, wildlife health, disease surveillance

## Abstract

There is an increasing need for robust wildlife health programs that provide surveillance and management for diseases in wildlife and wild aquatic populations to manage associated risks. This paper illustrates the value of a systematic method to enhancing wildlife health programs. The U.S. Geological Survey and Mahidol University, Faculty of Veterinary Science, Thailand National Wildlife Health Center formally twinned under the auspices of the World Organisation for Animal Health to enhance wildlife health capacity in Thailand and the Southeast Asia Region. We used a system-wide approach to holistically and interdependently enhance capacity. The project commenced with a wildlife health program needs assessment, and capacity enhancement focused on strengthening the general wildlife health surveillance network and improving wildlife health information management. Activities included partner surveys, interactive and didactic workshops, and individual personnel training. Topics included development of wildlife health information management systems, analysis of the current surveillance network, development of a Theory of Change for a strengthened surveillance network, planning workshops to create a wildlife health network, training on wildlife disease outbreak investigation and field sample collection, leading networks, and individual training on bioinformatics and laboratory techniques. Engagement of stakeholders at all levels, continuous communication throughout the project, use of both strategic planning tools and pedagogical methods, and using iterative and adaptive approaches, were key factors to the success of this project.

## 1 Introduction

Attention to wildlife health is greater now than it has been in decades, with international organizations encouraging nations to establish wildlife health programs to manage wildlife-associated risks (1). To support such efforts, adequate and appropriate knowledge, skills, commitment, structures, systems, partnerships, and leadership underpinning wildlife health programs are beneficial (2, 3). Design of a wildlife health program, therefore, requires a rigorous process to identify needs and resources, establish priority goals, and set out the processes needed to secure critical capacities to meet goals and objectives. Inadequate attention to program planning risks creating programs that provide an inadequate evidence base for decision making and resource allocation, and that may not be sustainable. In this paper, we use the creation of the Thailand Wildlife Health Network as a case study of a system-wide, collaborative approach for developing a wildlife health program.

Recent studies have identified an overall low level of capacity to perform wildlife disease surveillance, with marked variability between countries, illustrating the need for wildlife health program capacity enhancement (4). The World Organisation for Animal Health (WOAH) offers a Twinning Program with the goal to foster a more balanced geographical distribution of advanced expertise, allowing more countries to access high-quality diagnostic testing and technical knowledge within their own region (5). Each Twinning Project directly links an existing WOAH Reference Laboratory or Collaborating Center with a candidate institute wishing to improve its capacity and scientific expertise. Knowledge and skills are shared over a defined project period through staff exchanges, training of key personnel, etc. WOAH Laboratory Twinning Projects provide mutual benefits for both institutions, including the creation of joint research opportunities, the establishment of research networks, and sustainable capacity building. The aim is to support the application of the candidate institute to become a WOAH Reference Center, with benefits at country and regional levels.

During 2019–2024, the U.S. Geological Survey and Mahidol University, Faculty of Veterinary Science, Thailand National Wildlife Health Center formalized a Twinning arrangement under the auspices of WOAH to enhance wildlife health capacity in Thailand and the Southeast Asia Region. Capacity development is defined as “the process whereby individuals, organizations and society as a whole unleash, strengthen, create, adapt and maintain capacity to set and achieve their own development objectives over time” (6). We used a system-wide approach to holistically and interdependently enhance capacity to go beyond technical training, and we used participatory approaches to jointly assess capacity needs, to design context-specific capacity development activities, and to monitor results. The project encompassed the three dimensions of capacity enhancement, i.e., the enabling environment (institutional organization, the implicit and explicit rules, the power structures and the policy and legal environment in which individuals and organizations function); the institutional dimension (cross-sectoral, multistakeholder coordination and collaboration mechanisms; strategic management functions, structures and relationships; information, knowledge-sharing and decision-making processes; human and financial resources; and infrastructure); and the individual capacity (technical and functional knowledge, skills, competence levels, and attitudes of individuals). This paper outlines strategies to enhance wildlife health capacity through specific activities conducted in fulfillment of the Twinning Project. We discuss the key outcomes, their effectiveness, and lessons learned.

## 2 Methods

### 2.1 Project planning

The project's activities were informed by generic guiding questions for program planning (Table 1). Steps to answer these questions were woven throughout the activities described below. All efforts were taken to ensure that stakeholders and partners in the program were engaged, listened to, and incorporated into the planning process. We attempted to include a diversity of agencies, including ones that have historically been excluded from the One Health approach, such as the marine and zoo sectors. We used internal institutional knowledge, literature reviews, and information gathered from professional networks to select participants. We involved frontline workers, technical professionals, and leadership in the planning of the work. While the methods below suggest a linear process, this was an adaptively and iteratively managed process wherein what we learned from preceding activity informed the focus of subsequent activities. For example, the needs assessment revealed priority areas for subsequent situational assessment (e.g., wildlife health information management). Furthermore, insights and information gained in a subsequent activity would modify the interpretation of information gained from a previous activity.

**Table 1:** Guiding questions used to inform a system-wide wildlife health program capacity enhancement process in Thailand and activities undertaken to address these questions.

**Guiding questions**	**Activities to address the questions**
What will be the process for program development?	Creation of a project development steering group. Pre-project meetings to plan processes that are acceptable to stakeholders, feasible, and effective.
Why is this program needed?	Situational analysis and needs assessment. Developing mission and vision statements.
What must be done to meet the need?	Identify the scope of the program including geographic scope, populations of concern, roles and responsibilities, and outputs.
How will the program lead to outcomes that address the need?	Creation of program Theory of Change and logic models with explicit strategies, activities, outputs, outcomes, and impact. Prioritizing and planning to address critical gaps in skills, infrastructure, processes, resources, and partnerships.
Who must be part of the program to ensure it is effective, efficient, and acceptable?	Partner recruitment. Networking events to build shared visions and relationships. Collaborative leadership development.

### 2.2 Needs assessment

As a prelude to the project, a programmatic needs assessment was undertaken in October 2019 to identify gaps between “current state” and “future desired state.” The needs assessment used a systematic set of procedures to determine needs, examine their nature and causes, and set priorities for future action. The required functions and capacities of a wildlife health program were based on the publication by Stephen et al. (7), that used a literature review and consultation with subject matter experts to identify the core attributes of such programs, including diagnostic capabilities, applied epidemiology, health information management, harmonization and coordination, risk communication, applied research, disease management and health promotion, and program management and administration. To determine the current and future desired state, the assessors (Blehert, Sleeman, Stephen) reviewed national and program-level One Health, conservation, and disease management strategic and implementation plans. Prior assessments and prioritization studies were also reviewed. Semi-structured interviews with decision makers, stakeholders, and key staff were performed to gather information on immediate concerns related to wildlife-associated pathogens and understand perceptions on the value of wildlife disease surveillance and management. Information on the current functions and capabilities of the program, what skills and capabilities were needed in the future, priorities for surveillance and management, and obstacles to success were also gathered. Interviews focused on information within the scope of participants' professional roles and responsibilities, and no personal information was obtained. Visits to current facilities and important field sites were conducted to complete the picture regarding current capabilities and future needs.

In summary, the assessment identified two priority areas for capacity enhancement:

1) Development and implementation of a wildlife health data and information management system.
2) Enhancement of the general wildlife disease surveillance system (morbidity and mortality investigations) and formalization of the partner network.

### 2.3 Wildlife health information management

Due to the COVID-19 pandemic, the wildlife health information management capacity enhancement was conducted using a series of virtual workshops (November 2020 and March 2022; 54 participants each). Participants for all the workshops were selected from the networks of the Thailand National Wildlife Health Center and based on their subject matter expertise and professional responsibilities. Participants were from diverse sectors, and included the Thailand Departments of Livestock Development; National Parks, Wildlife and Plant Conservation; and Marine and Coastal Resources, Zoological Park Organization of Thailand, Mahidol University, and other academic institutions in Thailand. There were also representatives from the U.S. Centers for Disease Control and Prevention, and WOAH. For the first workshop, participants were provided background information on health information management, and then engaged in break-out, facilitated brainstorming sessions to gather information on current practices related to wildlife health information management. The following questions were explored:

1. How are data currently collected and stored? Is it effective? What are the risks of data loss? What is the ease of data use?
2. What are the main wildlife health surveillance objectives?
3. What are the different types of data that need to be collected in the system?
4. How will data be used? And in what format do data need to be accessible?
5. What are needs regarding data security, back up, access permissions, etc.?

The second multi-day workshop focused on codeveloping a conceptual model of a prototype information management system through facilitated discussions of the following questions:

1. What decisions regarding wildlife health need to be made?
2. What are the challenges related to wildlife disease/health surveillance and related information management?
3. What is the readiness of the wildlife health community?
4. What data streams currently exist?
5. What are the different data types collected as part of disease surveillance and how do they relate to each other?
6. What additional data streams would be useful?
7. Are there networks, relationships, and agreements to promote data sharing and management?
8. What is done to encourage timely and complete data entry, e.g., incentives, strategies?
9. What are the challenges related to data interoperability?
10. What information functions would be helpful to decision making (e.g., data visualization, data analysis and reporting, early alerts, data porting, etc.)?

### 2.4 General wildlife health surveillance network

This work commenced with a survey of stakeholders, using an online questionnaire distributed to 183 professionals (55.7% response rate) across Thailand working in wildlife, marine animal, livestock, domestic animal, zoo animal, environmental, and public health sectors. Twelve semi-structured interviews with key professionals were then performed and included both Thai and U.S. interviewers. The results of this survey have been previously published (8), and to summarize those results, sectors ranked disease control, early threat detection, identifying known or novel zoonotic diseases, and building trust as the most beneficial outcome of wildlife disease surveillance. Accessing data collected by one's own sector was identified as the most challenging yet least difficult to improve. Unclear legal authority was the second most frequently identified challenge. Interviewees explained that legal documentation required for cross-institutional collaborations posed a barrier to efficient communication and use of human resources. Survey respondents identified allocation of human resources, adequate budget, and having a clear communication system between sectors as highest priority areas for improvement.

This information was used to design an in-person workshop on enhancing general wildlife disease surveillance in Thailand which occurred in Pattaya, Thailand during June 2022 with 58 participants. Again, professionals from the same government sectors, non-governmental organizations, and academia were invited to participate. In addition, participants from USAID, the US Embassy in Thailand, and international experts from Laos attended.

The specific goals of the workshop were:

- Co-create a general wildlife disease surveillance plan for Thailand, including governance structure for a network and data sharing.
- Conduct a table-top exercise on a wildlife disease outbreak investigation in Thailand to evaluate current roles and responsibilities of various partners and identify potential preparedness needs.
- Develop logic models for enhancement of general wildlife disease surveillance system for Thailand, including current resources, lists of needed protocols/SOPs, and training needs to implement a general wildlife disease surveillance network.

The workshop consisted of plenary and breakout sessions using swim lane process map exercises (https://sixsigmadsi.com/what-is-a-swim-lane-process-map/) and logic models to elicit the desired information. Specifically, workshop attendees were asked to consider three scenarios occurring on a fictional national park, one involving detection of a wildlife-only pathogen, a second involving detection of agricultural pathogens, and third involving detection of a potential zoonotic pathogen. In small groups, the attendees worked together to create a process flow, or swim lane diagram of communication chain and actions among agencies and partners for the three scenarios. The groups also identified the authorities permitting sample collection and submission, intra- and inter-agency communication of diagnostic results, and how field and diagnostic information was currently stored and shared by partners. The diagrams were intended to delineate roles and responsibilities, show the connections, communication, and handoffs between the responsible parties to identify gaps, redundancies, and inefficiencies in the system.

The next step was to create a logic model/Theory of Change (9) for wildlife disease surveillance in Thailand through the development of a graphic illustration of the relationship between the resources, activities, and products and their relationship to the intended outcomes of the program. The information from the swim lane exercise was used to populate the current inputs, activities, and outputs for the Thailand general surveillance logic model, describing how current resources, activities and products were contributing to desired outcomes of general wildlife disease surveillance system in Thailand.

The workshop participants initially discussed the desired outcomes of a general wildlife disease surveillance program in Thailand. The goals identified during the Wildlife Health Information Management workshop were used to populate the outcomes for the program, and workshop participants identified enhancement of wildlife health and conservation and management of habitat and associated ecosystems as the primary impact of surveillance. In small group exercises, individual logic models were created for the five primary components needed for a general surveillance program: field detection network, laboratory network, information management, analysis and communication, and governance. The goal was to identify ways to overcome identified gaps using the logic models.

The workshop was followed by a meeting of 22 agency leadership and decision makers in Bangkok, Thailand during September 2022 to discuss the next steps in development of the wildlife health network, including policies needed to support such a network. Subsequently, a meeting was held in Bangkok, July 2023 to develop the strategic plan for Thailand's wildlife health network. The AIC method (Appreciation-Influence-Control) was used to collect the opinions and ideas from all participants (10). AIC is a whole-systems approach to stakeholder interaction, analysis, and collaborative planning. This method used brainstorming to understand the problem, limitations, needs, and potential contribution of stakeholders. The process was composed of three steps:

- Appreciate through listening: appreciate the realities and possibilities of the situation by gaining perspective on the stakeholders and situation.
- Influence through dialogue: explore the logical and strategic options for action as well as the subjective feelings and values that influence selection of strategies.
- Control through action: enable the stakeholders to take responsibility for choosing a course of action based on information discussed in the workshops.

The outcome of the workshop was the establishment of the network mission, vision, and objectives for senior leadership approval.

### 2.5 Core capacity development

The strategic planning workshops were complemented with technical training on topics identified as priorities during the discussions. This included a didactic and hands-on workshop for 45 technical experts and field staff in Bangkok during December 2022 on wildlife disease outbreak investigation, including field data and sample collection, biosafety, necropsy, communication, contingency planning, and diseases of different taxa. In addition, virtual workshops on network leadership were conducted during January 2023 for 22 staff responsible for the network management that included topics on building and sustaining a network, building support for the Theory of Change, leadership and governance for collective action, and inspiring support and action. Individualized training for staff from the Thailand National Wildlife Health Center on bioinformatics and rapid screening tests for wildlife and zoonotic diseases using monoclonal antibodies was also performed.

### 2.6 Evaluation and implementation planning

Finally, a closeout workshop in Bangkok in January 2024, and meetings with agency leadership were held to review progress and achievements and plan future actions. The workshop objectives included: present the project's results and main outcomes, gather partner feedback on the execution of the project, and co-create the next steps for implementation. Participants were asked for feedback on project satisfaction using a Mentimeter (Presentation Software, Stockholm, Sweden) survey. A Polak game assessment (11) was also performed to gather information on participants' perspectives on the future of wildlife health in Thailand, i.e., how optimistic were they and how much difference did they perceive they could make. In addition, participants were engaged in a discussion on the next steps for implementation and asked to rank the network objectives to determine priorities, using a simple scoring system in which each participant was given two votes for the four actions under each objective.

## 3 Results

### 3.1 Needs assessment

The needs assessment revealed well-developed diagnostic laboratories with capabilities to identify bacterial and viral pathogens using culture-based, serological, and molecular techniques; expertise in pathology; and ability to conduct live-animal studies. There were also good capabilities in biostatistics and epidemiological modeling. There was evidence of strong support from Mahidol University leadership for the program, and external stakeholders expressed support for the concept. The political support for the Thailand National Wildlife Health Center (via cabinet approval) suggested that Mahidol University had the necessary mandate to fulfill its mission. Finally, there was a good foundation of highly qualified personnel to deliver core services of a national center in place at Mahidol University.

Outbreak investigation capabilities were in the nascent stages of development. Field investigation capabilities resided with the Thailand Departments of National Parks, Wildlife, and Plant Conservation; and Marine and Coastal Resources, with a network of veterinarians. Capabilities in bioinformatics, data visualization and analysis were limited, and records were maintained in an Excel database. Standard approaches to data recording were not evident. There was a reliance on personal relationships to coordinate information and meet the expectations of partners rather than a systematic process, like an advisory committee or governance structure that outlined roles and responsibilities.

### 3.2 Wildlife health information management

The information management workshops revealed that most agencies and institutions maintained surveillance data; however, all current systems utilized paper or Excel spreadsheet tracking systems. The Department of Livestock Development maintained an online database, but it was not designed for wildlife health data. There was, however, consensus that the goals of surveillance data were to:

- Provide early detection of disease threats, including threats to wildlife, livestock, and humans. This information was also identified as important for directing disease control and prevention actions.
- Increase situational awareness, i.e., knowledge of current disease risks and assessment of these risks to wildlife, livestock, and people.
- Guide allocation of resources through knowledge of priority species, locations, and diseases.
- Improved understanding of disease dynamics (understanding the etiology of outbreaks and transmission) to direct management actions.
- Direct law enforcement actions.

Challenges identified were: Insufficient resources, including financial and human; lack of a centralized database and information management system; unclear authorities to take action; challenges related to intra- and inter-agency/organizational collaboration, including sensitivities of data sharing; differing agency priorities and rules related to data sharing; and poor sample quality due to sample deterioration, and lack of access to carcasses due to remote and challenging terrain.

The following factors were identified as positive attributes to the establishment of a general wildlife disease surveillance system and associated information management system:

- Thailand has good diagnostic laboratory capacity.
- Thailand Department of National Parks, Wildlife and Plant Conservation is currently using SMART Patrol for wildlife management (https://smartconservationtools.org/en-us/).
- Capacity of the academic sector is good.
- General willingness to coordinate and collaborate.
- Recognition of need for interoperability/data sharing, including with neighboring countries and regionally.

Agreed areas for improvement arising from the factors listed above were the need for: Improved coordination; addressing data sharing and privacy concerns through agreements; better integration of the wildlife/environmental sector into the One Health approach; electronically recorded data, and the creation of data integration systems to enhance data sharing.

**Figure 1:**
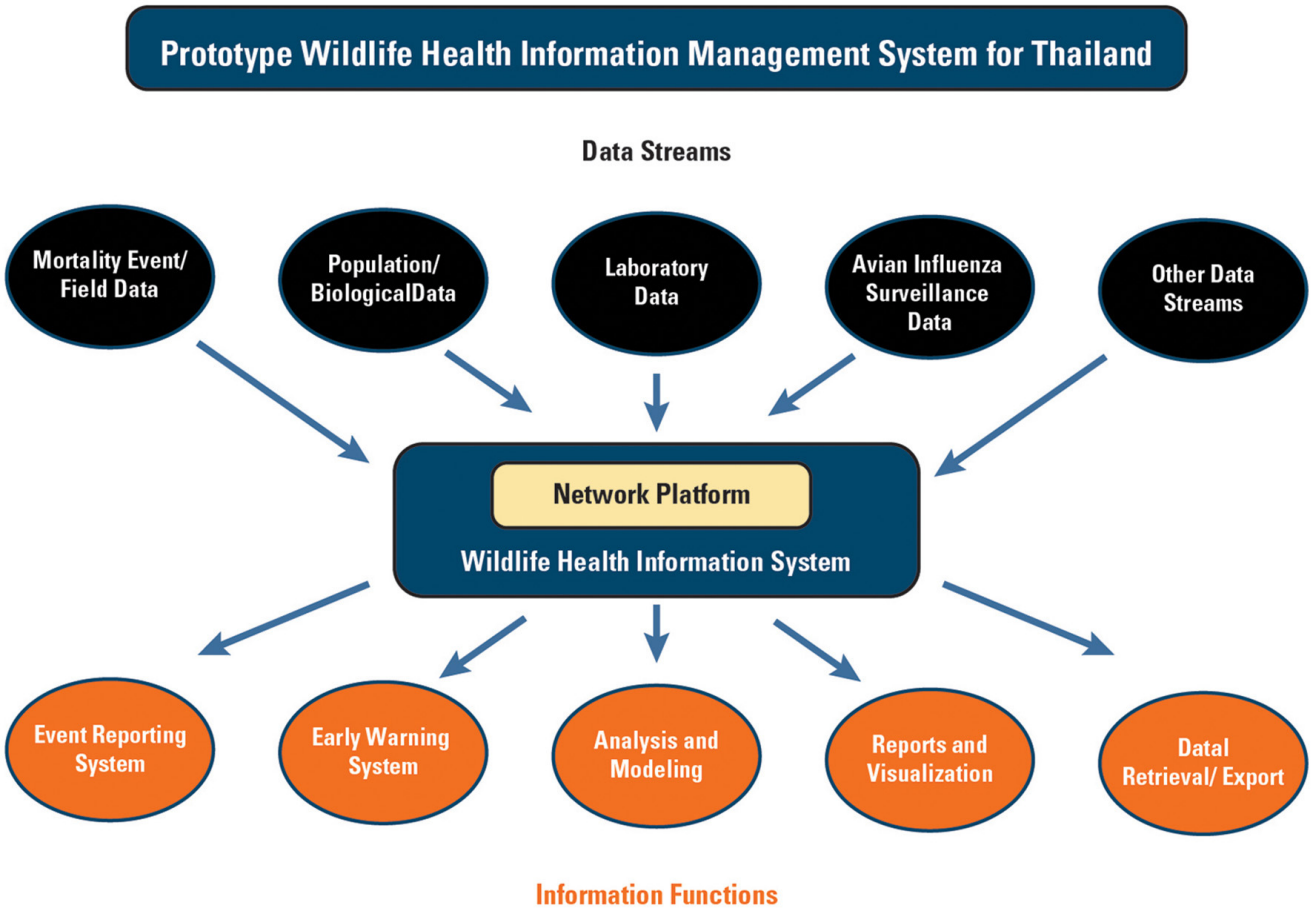
Potential conceptual, user defined wildlife health information management system for Thailand (color).

The desired data streams and data fields were identified and listed in Table 2. The workshops concluded with development of a health information management design concept (Figure 1). There was consensus that web-based reporting would be highly desirable with different levels of access for different users. Near real-time web-based mapping of diseases was also identified as desirable. Other desired functions included a data dashboard, the ability to export data in different formats, near real-time data reporting and alerts when diseases of interest have been identified, display of wildlife population distribution and migratory movements; display of agricultural animal and human population distributions, summary (infographic) reports, and sharing of information internationally to WOAH.

**Table 2:** The identified types of data streams and data fields for a conceptual wildlife health information management system for Thailand.

Disease investigation data, including case history, environmental data
Laboratory data, including microbiology and pathology data, and next generation/whole genomic sequencing data
Wildlife population data (species range, migration, demographics, etc.)
Environmental data, including habitat quality, climate, weather, etc.
Data on distribution of invasive/alien species
Human and livestock demographic data
Information from local communities
Socio-political data

### 3.3 General wildlife disease surveillance network

**Figure 2:**
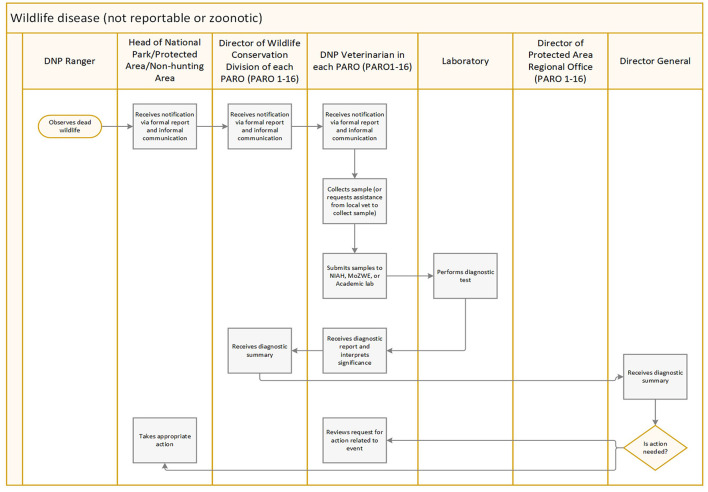
Process flow diagram for a wildlife disease outbreak investigation in Thailand. The laboratory performing diagnostics during an investigation is determined by proximity, veterinarian relationships with academic laboratories, diagnostic test capabilities of the laboratory, cost, and whether confirmation will be needed for a test result (as is the case for WOAH listed diseases). When there is media interest in an event the Regional Veterinarian from DNP may accompany the Chief Veterinarian during sample collection. DNP, Thailand Department of National Parks, Wildlife, and Plant Conservation; PARO, Protected Area Regional Office.

The swim lane exercises revealed that when a wildlife health event occurred in a national park, communication followed a hierarchy from observation of the event made by a Department of National Parks, Wildlife and Plant Conservation (DNP) park ranger through successive managers up to the Director General (Figure 2). The Director General was responsible for the decision to take action during an event. Communication during an event was both official (mailed paper reports and memos) and informal (messaging applications, phone, and email). Epidemiological information (species affected, number affected, location, etc.) about an event occurring within a national park was typically recorded on paper and diagnostic samples were collected by a DNP veterinarian. However, depending on the location of the event, the species involved and the suspected pathogen, a Department of Livestock Development or local veterinarian may do an initial site inspection and determine if samples can be collected prior to a site visit by the DNP veterinarian. Diagnostic results were stored in excel spreadsheets or a laboratory information management system (LIMS), depending on the laboratory where tests were performed. The laboratory performing diagnostics during an investigation was determined by proximity to the event, DNP veterinarian's relationships with academic laboratories, diagnostic test capabilities of the laboratory, cost, wildlife species involved, and whether confirmation will be needed for a test result (as is the case for WOAH notifiable diseases). When an agricultural pathogen was suspected in wildlife the Department of Livestock Development was included in the communication chain and during sample collection. When a zoonotic pathogen was suspected in wildlife, the Department of Disease Control was included in the communication chain and during the sample collection. When a wildlife morbidity or mortality event was investigated in aquatic species, the Department of Marine and Coastal Resources conducted the investigation.

Several impediments that could negatively impact implementation of a general wildlife disease surveillance program were identified and grouped into three main categories: (1) the need for a governance structure to support the administration, coordination, and functioning of a national wildlife disease surveillance system, (2) the lack of an information management system and centralized database; and (3) the lack of laboratory coordination and standardization in some cases along with inconsistent sample quality and quantity.

**Figure 3:**
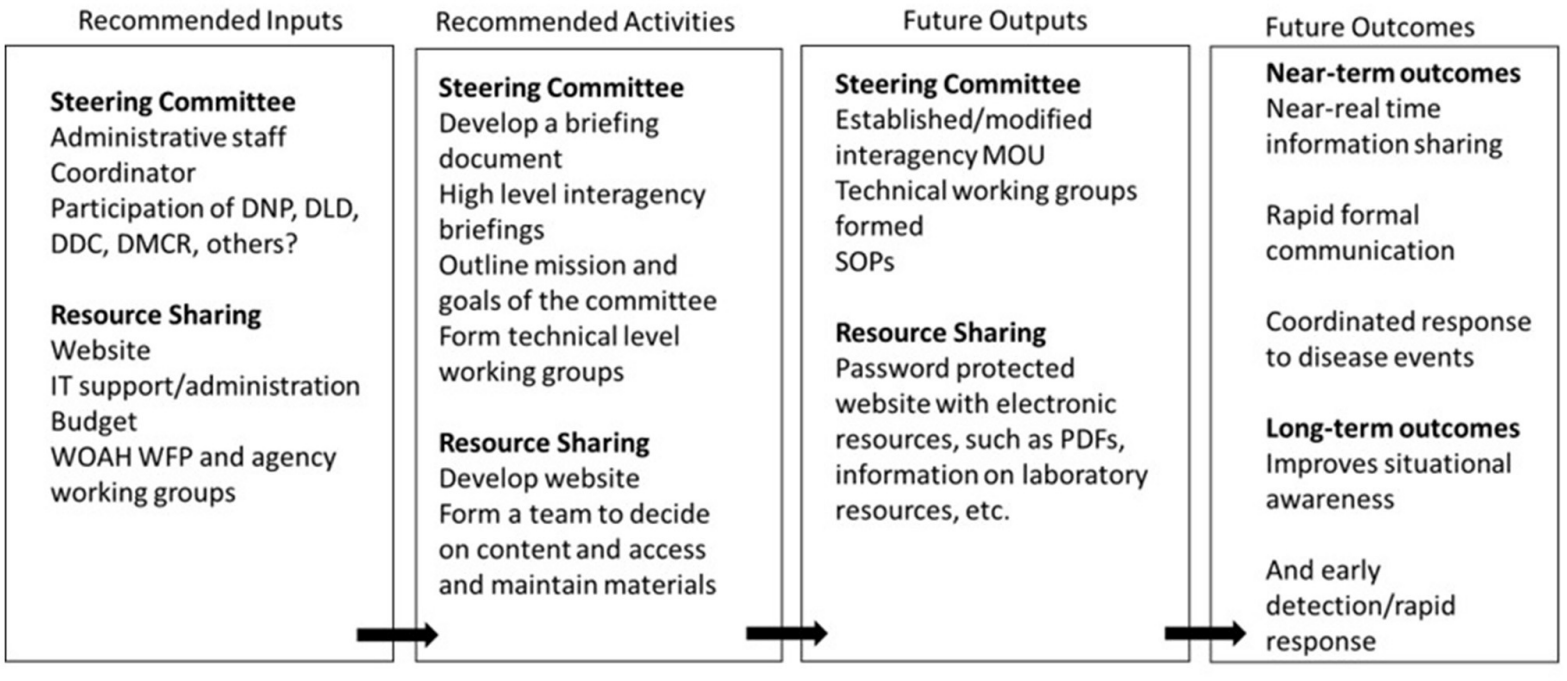
Logic model of the needed resources (inputs), future activities, products (outputs), and outcomes recommended by workshop participants for coordination and governance of a general surveillance program in Thailand.

An example logic model is provided in Figure 3 and represents the steps needed to fill the gaps of the resources (inputs) and activities (outputs) that could enhance the effectiveness of Thailand's general surveillance program. The key outcomes identified were early detection of wildlife health events in Thailand and improved situational awareness of wildlife health. The logic models also identified the following training needs:

- Wildlife disease outbreak investigation and disease control and prevention.
- Surveillance design and data management.
- Training on coordination and communication (network management).

Additional key recommendations from the logic models were the need for standard operating procedures (SOP), diagnostic standards, case definitions, formation of a governance structure such as steering committee, agreements such as memoranda of understanding (MOU) developed jointly with all partners participating in the national surveillance for wildlife diseases, and development of a national policy to ensure sustainable support for a national general wildlife disease surveillance system. Based on discussions among the workshop participants, the priority next steps identified were:

- Formation of a national level policy that would ensure sustainable resources and, concurrently, the creation of a wildlife disease surveillance steering committee and associated national wildlife health surveillance strategy.
- Explore piloting a wildlife disease data and information management system.
- Creation of a web-based information resource center where disease plans, SOPs, field manuals, information on diagnostic laboratories, and other information deemed useful by the community can be shared.

**Figure 4:**
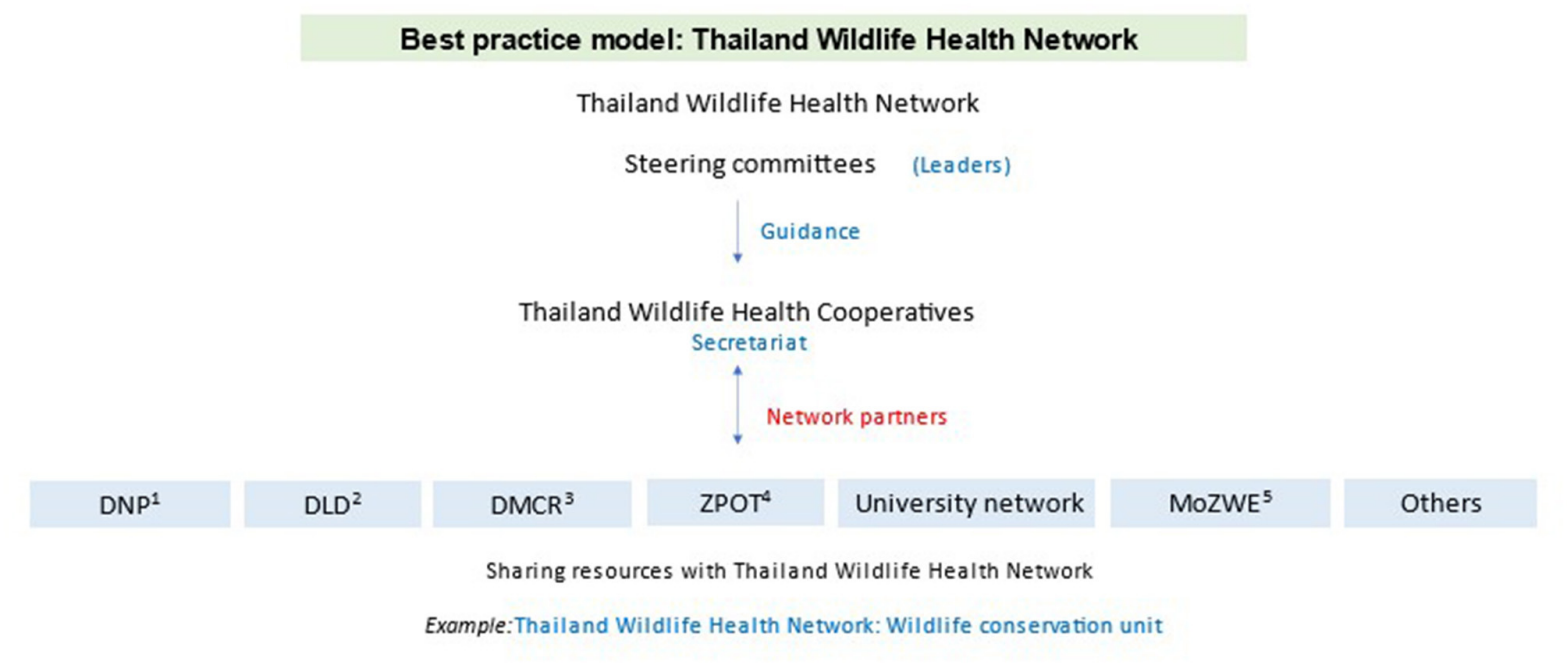
Potential governance and structure for a wildlife health network in Thailand (color). DNP, Thailand Department of National Parks, Wildlife, and Plant Conservation; DLD, Thailand Department of Livestock Development; DMCR, Thailand Department of Marine and Coastal Resources; ZPOT, Zoological Park Organization of Thailand; MoZWE, The Monitoring and Surveillance Center for Zoonotic Diseases in Wildlife and Exotic Animals, Faculty of Veterinary Science, Mahidol University.

At the high-level, agency leadership meeting in September 2022, participants agreed to and proposed a Thailand Wildlife Health Network model (Figure 4). The Thailand National Wildlife Health Center would act as a secretariat to facilitate the connection among the focal points and network, and a steering committee consisting of agency leadership would oversee the secretariat and approve network policies and procedures. Each participating agency or organization would nominate a focal point who would participate in the regular meetings to increase trust and share information among partners.

At the strategic planning meeting in the summer of 2023 three strategies were agreed upon to support the network; (1) strengthen the network through clarification of roles, responsibilities, and operating procedures, and establishment of interagency MOUs and data sharing agreements; (2) enhance knowledge through continuing professional development; and (3) sustain the network by developing the wildlife health information system. These strategies would support the mission of the network to support and facilitate wildlife disease surveillance, early threat detection, and research on wildlife and ecosystem health.

### 3.4 Closeout workshop

At the closeout workshop in January, 2024, 87% of the participants indicated that the project had met or exceeded their expectations. In addition, 74% of the participants self-scored as in the powerful quadrant of the Polak matrix, i.e., they believed that the situation was good and believed that it can get better and were confident in their ability to act and create a better future. No one scored themselves in the powerless quadrant. Priorities for implementation of the network that were ranked highest by the participants were drafting a charter and implementation document, outlining network function and roles and responsibilities; establishment of the network governance; and development of SOPs, guidelines, and protocols.

## 4 Discussion

We describe the methods and outcomes of a multi-year wildlife health capacity enhancement project following the WOAH Twinning process Program that successfully created a formalized national wildlife health network and enhanced technical knowledge in wildlife disease surveillance and network management. Despite the identified need to enhance global capacity in wildlife disease surveillance, there are few previously published studies on this topic. Valeix et al. (12) used interviews to assess the feasibility of establishing a National Wildlife Health Center in Sri Lanka. Pruvot et al. (13) described a locally-driven, One Health approach to establishing wildlife health surveillance in Cambodia, Lao Peoples Democratic Republic, and Viet Nam under the WildHealthNet initiative. They identified cross-sectoral and trans-disciplinary approaches as critical to success. Further, long-term commitment, and paralleled implementation and policy development was identified as key to sustainable wildlife health surveillance. Unwin et al. (14) evaluated the success of capacity enhancement activities for the Orangutan Veterinary Advisory Group that were guided by a Theory of Change and found significant improvement in skills. Our project integrated these methods and additional ones to deliver a comprehensive, multi-strategy capacity enhancement project. Taking the system-wide approach allowed for the enhancement of both technical and functional capacity, including development of leadership skills, increased capacity for collective action, institutionalization of life-long learning, and engagement in policy and planning processes. We believe that engagement of stakeholders at all levels, from frontline workers, technical professionals, and decision makers, continuous, bi-directional communication throughout the project, use of both strategic planning tools and pedagogical approaches, and using iterative (gathering the same information using different techniques) and adaptive (modifying the training based on information gathered) approaches, were key factors to the success of this project.

The needs assessment proved to be an effective and efficient method to determine priority programmatic gaps and co-develop next steps. The priority needs of enhancing and formalizing a wildlife health network and developing an associated information management system were identified as priorities through the surveys. The swim-lane and logic model exercises corroborated the needs assessment findings. Consequently, wildlife health capacity enhancement projects may benefit from commencing with a needs assessment or gap analysis to maximize the use of resources and impact.

The virtual information management workshops were successful in defining the user requirements of the system, i.e., they provided a description of what the system should do, the service or services that it should provide, and the constraints on its operations. However, they were not adequate to address the more complicated topics such as data sharing agreements. Thus, further development of the system was placed on hold and integrated into the wildlife health network goals and objectives. While this project showed that significant insights can be garnered from virtual meetings, discussion of complex and sensitive topics in this case would have benefited from in-person meetings and discussions.

The swim-lane exercise revealed that the current system in Thailand of general wildlife health surveillance may benefit from less bureaucracy, and increased efficiency and coordination. Formation of a wildlife health network was identified as a priority action to enhance the system, which was consistent with the findings in the survey questionnaire (8). Intriguingly, participants in the survey identified building trust as a benefit of wildlife health surveillance, which augurs well for the success of the network, and is consistent with other similar surveys (15). In situations where there are multiple partners with different missions, unclear legal authorities, and sensitivities such as data sharing, a network may help to enhance collaboration, increase coordination, resolve conflicts, and build trust. Recognizing the centrality of the network to the success in enhancing wildlife health surveillance, we added training on network leadership and management as described to help ensure the network is well managed and there is transparency, equity, and mutual benefit in the network. Including training on “softer” skills such as leadership in addition to the technical skills may be helpful in wildlife health capacity enhancement projects.

Limitations of this approach included the intermittent nature of the workshops over several years. This required repeated engagement with agency leadership to ensure awareness of the project and understanding of the value due to personnel changes. A consistent on-site presence in Thailand may have been beneficial in ensuring continuous engagement and building trust; however, this would have increased the project costs.

Despite this, a majority of participants believed that the project met or exceeded expectations and felt optimistic about the future and able to contribute. This indicates that the process undertaken to plan the development of the Thailand Wildlife Health Network was successful in achieving the goals, and augurs well for further development and sustainability of wildlife health program in Thailand. This work has also resulted in a sustainable Wildlife Health Network for Southeast Asia that Mahidol University serves as the secretariat for, further illustrating the success of the project. Future planned activities include high-level meetings with leadership and decision makers to continue to gather support for the network, funding raising for the wildlife health information management system, development of a wildlife health information clearinghouse, and continued collaboration between USGS and Mahidol University. Repeating the needs assessment in 3–5 years could be helpful in further documenting any improvements to the program.

Using a system-wide approach ensured that the capacity enhancement work was co-created and context-specific from the outset of the project and focused on the priority needs. We believe that enhancing institutional capacity and the enabling environment, in addition to individual and technical training, can help ensure the success and sustainability of the outlined activities. Relationships and trust between organizations were developed through continuous, collaborative, inclusive, and equitable engagement in training and planning to ensure mutual benefit. We believe the project benefited from the strategic planning methods used including stakeholder interviews, surveys, gap assessments, and logic models. The participatory approach was vital to development of the Theory of Change that helped identify core inputs and activities to achieve the desired outcomes and to help explain the network to potential partners and supporters. Other wildlife health capacity enhancement projects may benefit from a similar approach.

## Data Availability

The original contributions presented in the study are included in the article/supplementary material, further inquiries can be directed to the corresponding author.
